# Selected Spermatozoa at Conventional Magnification Cannot Guarantee in Obtaining Spermatozoa With Long Telomere Length in Severe Teratozoospermia Patients

**DOI:** 10.7759/cureus.77240

**Published:** 2025-01-10

**Authors:** Fatemeh Shakeri, Ali Nabi, Ehsan Farashahi, Saeideh Erfanian, Azam Agha-Rahimi

**Affiliations:** 1 Reproductive Biology, Research and Clinical Center for Infertility, Yazd Reproductive Sciences Institute, Shahid Sadoughi University of Medical Sciences, Yazd, IRN; 2 Stem Cell Biology, Stem Cell Biology Research Center, Yazd Reproductive Sciences Institute, Shahid Sadoughi University of Medical Sciences, Yazd, IRN; 3 Tissue Engineering, Faculty of Basic Sciences and Advanced Technologies in Medicine, Royan Institute, Academic Center for Education, Culture, and Research (ACECR), Tehran, IRN

**Keywords:** density gradient centrifugation, dna fragmentation, spermatozoa, telomere, teratozoospermia

## Abstract

Background

Sperm selection from the population of processed spermatozoa cells after density gradient centrifugation (DGC) can assist embryologists in selecting high-quality sperm. Sperm selection of low-quality and chromatin-damaged spermatozoa is inevitable in severe teratozoospermia semen specimens. This study was conducted to evaluate whether sperm selection at ×400 magnification enables embryologists to select a population of spermatozoa with low DNA fragmentation and high sperm telomere length (STL) in semen samples with severe teratozoospermia.

Methods

A total of 23 infertile men characterized by severe teratozoospermia were selected. Sperm DNA fragmentation (SDF) and relative STL (r-STL) were evaluated at three stages: specimen collection, after DGC, and during the single selection of spermatozoa at ×400 magnification (single selection). The 23 patients were divided into two groups, including 14 with normal morphology ≤1% and nine with normal morphology of 2%. SDF and r-STL were compared between the two groups at three stages.

Results

The results of this study showed that although SDF decreased remarkably after DGC and single selection (F=64.327, P-value=0.000), the DNA fragmentation index obtained for each semen sample was more than the cutoff point of 18% based on the Halo sperm test. No statistically significant differences were observed in r-STL after DGC and single selection (F=1.978, P-value=0.163). Meanwhile, the pairwise comparison of r-STL showed that in the 2% normal morphology group, the mean relative telomere length was significantly higher in the selected spermatozoa compared to the semen specimen (P=0.014). This increase can be attributed to DGC and single selection by the embryologist. Also, there was no correlation between SDF and r-STL in the semen samples with severe teratozoospermia (r=0.01, P-value=0.964).

Conclusions

This study suggests that investing more time in sperm selection can decrease SDF, but r-STL of spermatozoa selected by the embryologist does not increase in severe teratozoospermia semen samples with morphology ≤1%.

## Introduction

Male factor infertility is considered the sole cause of infertility in 30% of infertile couples, and a contributing factor in an additional 20%, joined with female factor infertility [1]. Although semen analysis is the foundation of male fertility assessment, this microscopic method does not provide real discrimination between fertile and infertile men [2]. In addition to routine semen analysis, sperm DNA fragmentation index (s-DFI) measurement can present additional information on sperm quality in the assessment of infertile couples with male factor infertility [3,4].

Several studies have shown that the reactive oxygen species level is higher among infertile men than fertile men, negatively impacting proper DNA packaging and leading to higher DNA fragmentation [5], which is followed by poor embryo development, lower implantation rate, and higher miscarriage rate [3]. Oxidative stress can lead to chromatin packaging damage and alter telomeric distribution and length [6,7] Also, the enrichment of telomere sequences with guanine repeats is another potential factor increasing sensitivity to oxidative stress [8].

Telomeres are characterized by non-coding DNA sequences at the end of the chromosomes. They play a critical role in the maintenance of genomic integrity and stability. Telomere sequences are localized peripherally, attached to the sperm nuclear matrix [9]. As a result of telomere shortening, telomere sequences lose their connection to the nuclear matrix and fertilization fails [7].

In clinical intracytoplasmic sperm injection (ICSI), embryologists try to select motile spermatozoa with normal morphology to inject an oocyte. Among semen parameters, morphology and motility are considered two predictive factors in terms of DNA fragmentation and sperm telomere length (STL) [6,10]. Also, single-cell assays of telomere length among spermatozoa within an ejaculate sample showed an increased heterogeneity of STL, therefore, sperm selection based on morphology can be a promising tool for selecting spermatozoa with normal telomere length [11,12]. The selection of motile spermatozoa with normal morphology at ×400 magnification can thus help embryologists choose spermatozoa with low DFI and high STL, thereby improving the fertilization rate [7], embryo development, and pregnancy outcome [13]. However, Avendaño et al. (2009) detected DNA fragmentation in morphologically normal spermatozoa fraction of semen samples obtained from patients with moderate to severe teratozoospermia [14].

Sperm selection under conventional magnification is a time-consuming procedure that requires extensive labor and precision by the embryologist, especially in patients with severe teratozoospermia. Sperm with proper morphology is defined as oval-head sperm without nuclear vacuoles, but in teratozoospermia samples, the selection of low-quality and chromatin-damaged spermatozoa is inevitable [15]. This study was designed to assess whether patients with severe teratozoospermia can benefit from sperm selection at ×400 magnification.

The review of the literature did not reveal any research examining whether sperm selection based on motile spermatozoa with normal-looking heads can help embryologists select spermatozoa with high STL and low DNA fragmentation in semen samples with severe teratozoospermia. Therefore, the present study was conducted on infertile couples with male factor infertility characterized by severe teratozoospermia to evaluate their STL and sperm DNA fragmentation in three stages including raw semen samples, the population of spermatozoa after DGC, and the spermatozoa selected individually by the embryologist at ×400 magnification.

## Materials and methods

Study design and participants

This study was conducted on infertile men with severe teratozoospermia among the infertile couples attending the Research and Clinical Center for Infertility, Yazd Reproductive Sciences Institute, from January to May 2024. Written consent was obtained from the infertile men and the surplus of their semen specimens was used. The Ethics Committee of Shahid Sadoughi University of Medical Sciences, Yazd issued approval IR.SSU.MEDICINE.REC.1400.308, dated December 18, 2021. The population of the study consisted of 23 infertile men by considering a power of 90%, ɑ=0.05. This sample size was calculated from a previous study, which evaluated sperm telomere length in 15 infertile men with normozoospermic features [16].

The sperm samples from the male participants were characterized by fresh ejaculated spermatozoa with a concentration of at least 3 million/ml and had an indication for ICSI due to male infertility. The infertile men were undergoing conventional semen analysis and morphology assessment with the Diff-Quik test. The semen samples characterized by severe teratozoospermia were entered into the study. Severe teratozoospermia was defined as a morphology index of 0-2 [17]. The assessment of SDF and STL was performed in three stages for each patient: raw semen, in the processed sample after DGC (processed sample), and the selected spermatozoa as selected by an expert embryologist at ×400 magnification.

Sperm selection at ×400 magnification was performed by an embryologist with expertise in sperm selection in a clinical setting. The embryologist selected the motile sperms that could be injected during the ICSI procedure. For harvesting the selected spermatozoa, an ICSI dish (Falcon, Corning, New York, USA) was prepared, which contained oval-shaped droplets of polyvinyl pyrrolidone (PVP) 7% (Cooper Surgical, Melville, New York, USA), and 1 µl droplet of SynVitro Flush (Orgio, Måløv, Denmark) was placed opposite PVP droplet. The droplets were covered by 1.5ml light mineral oil to prevent evaporation. One µl of the processed sample was loaded on the left side of the 5µl droplet of PVP. Sperm selection was performed at ×400 magnification. The selected sperms were divided into two portions. One portion was used to evaluate sperm DNA fragmentation using the sperm chromatin dispersion (SCD) test. The second portion was used to evaluate STL by quantitative polymerase chain reaction (qPCR).

Sperm analysis and morphological assessment

Semen samples were obtained by masturbation after two to seven days of abstinence. The sperm concentration, motility, and morphology were determined based on the 2021 WHO guidelines, after liquefaction for 30-60 min at 37˚C [2]. Morphology was evaluated by the Diff-Quik rapid staining kit (IVF Co., Tehran, Iran) according to the manufacturer’s instructions and the sperm was processed by DGC at 300 g for 15 min. Semen parameters were assessed in 200 spermatozoa cells for each sample according to the 2021 WHO guidelines [2].

Sperm chromatin dispersion assay

Sperm DNA fragmentation (SDF) was evaluated using the Halo sperm kit (Sperm DNA Fragmentation Assay Kit, IVF Co., Tehran, Iran), according to the manufacturer’s instructions for the raw semen specimen and processed samples. The instructions for this step have previously been described by Anbari et al. (2021) [18]. The single selected sperms were aspirated into an injection needle and immersed into 4 µl of low-melting agarose on a pre-coated glass slide under ×200 magnification with an inverted microscope. DFI was evaluated based on the presence and the size of the halo under light microscopy. Large -and medium-sized halos were taken as sperm with no DNA fragmentation while small-sized or no halos were identified as sperm with fragmented DNA. DFI was calculated by counting 200 spermatozoa for the raw semen specimen and the prepared sample.

Sperm telomere length measurement

Genomic DNA was extracted from the semen specimen, processed samples, individual spermatozoa, and blood samples by DNJia Micro Kit (ROJE Technologies, Tehran, Iran) (#DN013070) following the manufacturer’s instructions. Relative STL and leukocyte telomere length were measured by qPCR according to Joglekar’s protocol [19] with some modification, where 36B4 was used as the single-copy gene. The primer sequences are listed in Table 1.

**Table 1:** List of primers used in the measurement of sperm telomere length. Source: Cawthon RM (2002) [20].

Primer	Sequence
Telomere-F	5′-CGGTTTGTTTGGGTTTGGGTTTGGGTTTGGGTTTGGGTT-3′
Telomere-R	5′-GGCTTGCCTTACCCTTACCCTTACCCTTACCCTTACCCT-3′
36B4-F	5′-CAGCAAGTGGGAAGGTGTAATCC-3′
36B4-R	5′-CCCATTCTATCATCAACGGGTACAA-3′

All the samples were run triplicate, and 5 ng of DNA per µl in a 10 final reaction volume was used for each PCR reaction. The average cycle threshold (Ct) and standard deviation of each sample, no template control (NTC), and leukocyte (Control) DNA were calculated, and the ∆Ct for each sample was determined as: average Ct of 36B4 - average Ct of telomere. ∆∆Ct was defined as: ∆Ct of each sample - ∆Ct control. This value was presented as the relative telomere length (RTL) [20].

Statistical analysis

The statistical analysis of the data was performed in IBM SPSS Statistics for Windows, Version 20 (IBM Corp., Armonk, NY, USA). The normality distribution of the basic characteristics of the patients was evaluated by the Kolmogorov-Smirnov test. The descriptive results were presented as mean ± SD or median and interquartile range (IQR). Normality checks were carried out on the SDF data at three stages showing normal distribution. A repeated-measures ANOVA was performed for the pairwise comparisons of SDF within each group.

Mann-Whitney’s U Test was performed for the comparative evaluation of STL between the two groups. Pairwise comparisons of STL within each group were performed using related samples of Friedman’s Two-Way Analysis Variance by Ranks. The data graphics were drawn using GraphPad Prism software, version 10.1.2 (GraphPad Software, San Diego, CA, USA). A P-value of 0.05 was considered statistically significant.

## Results

Characterization of patients and samples

The study was conducted on 23 infertile couples with severe male factor infertility. According to the WHO criteria, four patients were characterized as severe teratozoospermia, 16 as astenoteratozoospermia (AT), and three as oligoastenoteratozoospermia (OAT). The basic characteristics of the 23 patients are shown in Table 2.

**Table 2:** Basic characteristics of patients with severe teratozoospermia. DFI: DNA fragmentation index.

	Minimum	Maximum	Mean ± SD
Age (year)	27	51	35.17 ± 5.68
BMI (kg/m^2^)	18.37	40.91	27.58 ± 5.41
Concentration (10^6 ^sperm/ml)	5	65	29.34 ± 17.34
Progressive motility (%)	10	37	23.52 ± 6.74
Immotile (%)	53	80	66.13 ± 7.25
DFI of semen (%)	22	39	27.73 ± 4.28

**Figure 1:**
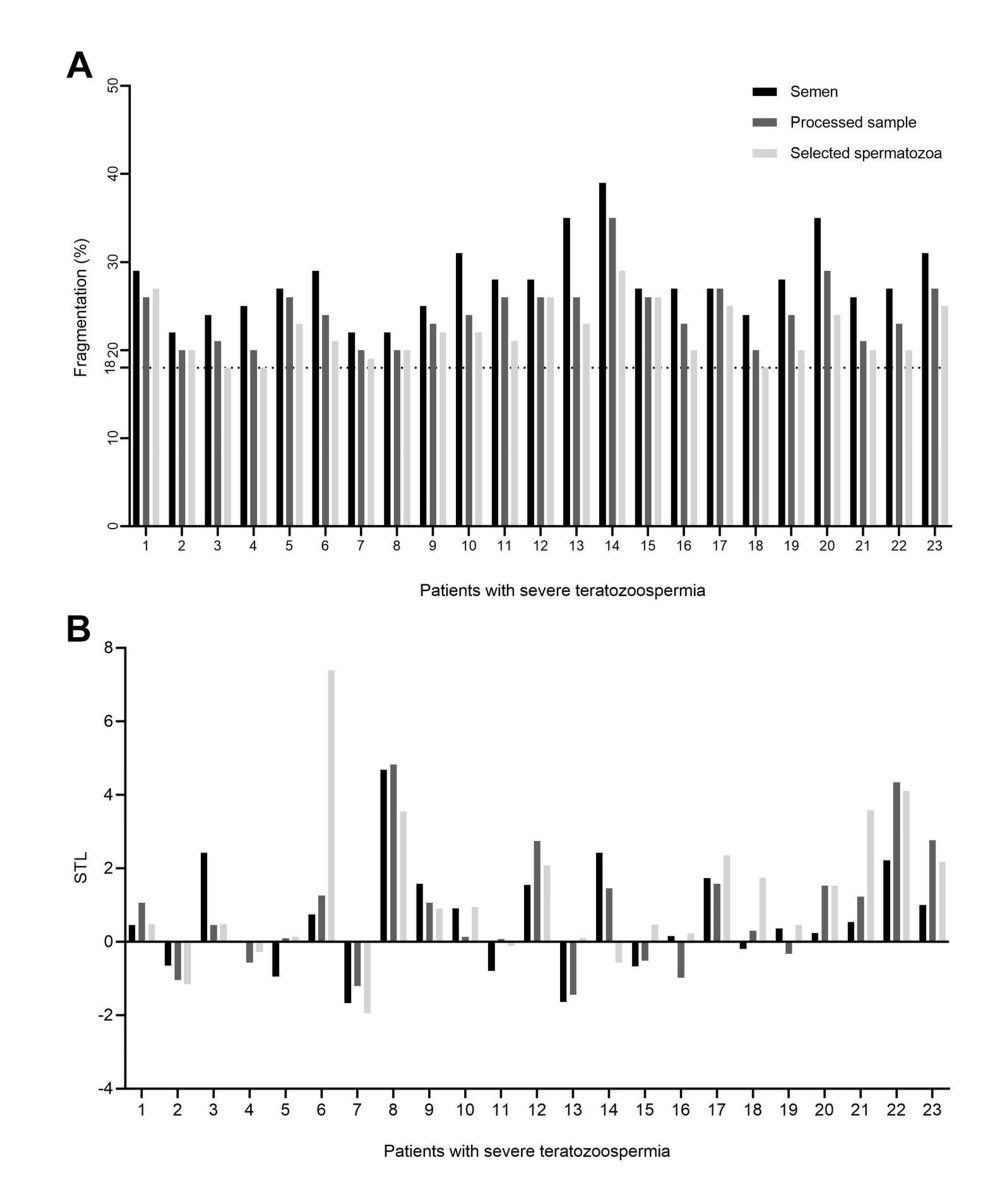
Evaluation the percentage of SDF (A) and relative STL (B) of 23 infertile men after each selection procedure. (A) Percentage of sperm cells with fragmented DNA in 23 sperm samples before and after DGC and population of single selected spermatozoa cells at ×400 magnification. (B) Relative sperm telomere length of 23 infertile patients before and after DGC and selected spermatozoa cells at ×400 magnification by an embryologist. SDF: sperm DNA fragmentation, STL: sperm telomere length, DGC: density gradient centrifugation.

The level of each SDF and RTL alteration was evaluated (Figures 1A, 1B). In the next step, samples were divided into two groups according to morphology index. Morphology indexes were reported as 2% or as 1% and less than 1%. The data about 1% and less than 1% were reported as one group (morphology ≤1). Of all 23 patients, 14 patients were entered in the morphology ≤1 (Group 1) and nine in the morphology of 2% (Group 2). Therefore, comparative evaluations were performed between the two-morphology categorization. No statistically significant differences were observed between the two morphologies with respect to age and Body Mass Index (BMI) at baseline (P= 0.305 and P= 0.688, respectively). Also, there were no remarkable differences between morphology ≤1% and 2% in terms of semen parameters (concentration: P=0.249, progressive motility: P=0.355, non-progressive motility: P=0.925, immotile sperm: P=0.375).

Comparative evaluation of sperm DNA fragmentation after each selection procedure

**Figure 2:**
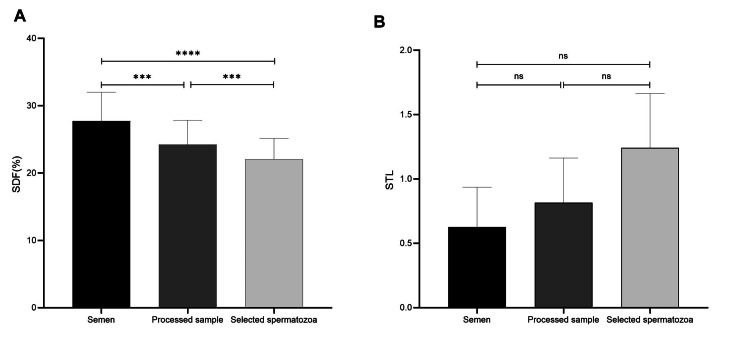
Evaluation of sperm DNA fragmentation (A) and relative STL (B) after each selection procedure. (A) Comparative evaluation of SDF in 23 patients characterized by severe teratozoospermia before and after DGC and selected spermatozoa at ×400 magnification by an embryologist. Analysis was performed with repeated measures ANOVA. (***indicates P˂0.001, ****indicates P˂0.0000.) (B) Comparative evaluation of relative STL in 23 patients between semen, prepared sample with DGC, and single selected spermatozoa at ×400 magnification by an embryologist. Analysis was performed with repeated measures ANOVA. (ns: P˃0.05; F=1.978, P-value=0.163.) SDF: sperm DNA fragmentation, STL: sperm telomere length, DGC: density gradient centrifugation, ns: non-significant.

The optimal value of 18% measured by the Halo sperm test is considered the cut-off for SDF to distinguish between men with and without teratozoospermia [21]. As shown in Figure 1, the DFI of each semen sample was more than the cut-off point after omitting the dead spermatozoa by DGC and single sperm selection. The mean DFI of the 23 male patients at baseline (raw semen specimens), processed samples (after DGC), and after single selection at ×400 magnification by the embryologist were calculated to be 27.73 ± 4.28, 24.21 ± 3.61 and 22.04 ± 3.11, respectively. SDF dropped by approximately 12.7% and 8.97% with DGC and single selection by the embryologist at ×400 magnification, which are significant numbers (P=0.000, P=0.000) (Figure 2A). 

**Figure 3:**
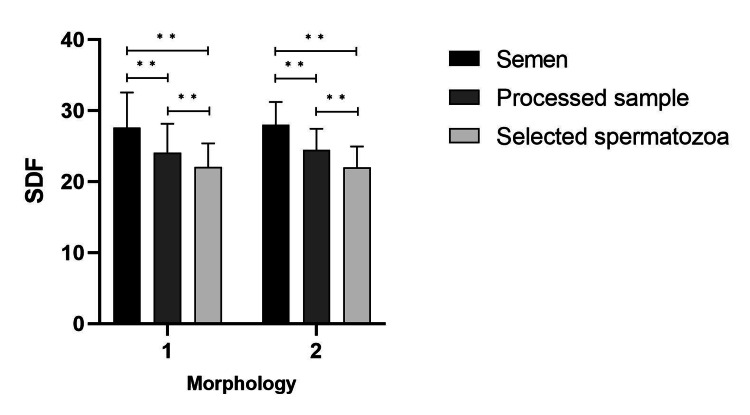
Pairwise comparison of SDF within each group of morphology. Pairwise comparison of SDF within each morphology was statistically significant. Analysis was performed with repeated measures ANOVA. (**indicates P˂0.01.) SDF: sperm DNA fragmentation, DGC: density gradient centrifugation.

As shown in Table 3, there was no statistically significant difference between the two sub-divided morphology in terms of SDF. The repeated-measures ANOVA with Bonferroni correction showed that the mean SDF differed significantly between the two procedures; after DGS and with single selection by the embryologist (F=59.91, P=0.001) (Table 3). The pairwise comparison of SDF was performed within each group to evaluate which procedures decreased SDF (Figure 3). As shown in Table 3, there were no statistically significant differences between the two morphologies in terms of SDF.

**Table 3:** Comparative evaluation of DGC and single selection of spermatozoa on SDF between two morphologies. ^a^Statistical analysis was done based on repeated measures ANOVA. P<0.05 is considered statistically significant. Bonferroni was used to adjust for multiple comparisons within each group. ^*^Processed samples: Semen samples after DGC, ^#^Single selection: Population of spermatozoa cells selected under ×400 magnification by an embryologist. SDF: sperm DNA fragmentation, DGC: density gradient centrifugation.

Morphology					
SDF/group	Group 1	Group 2	P-value	Procedure effect	Procedure group effect
	Mean ± SD	Mean ± SD			
SDF semen	27.54 ± 4.97	28.00 ± 3.20	0.821	F=59.91, P=0.001^a^	F=0.134, P=0.805^a^
SDF processed sample^*^	24.07 ± 4.06	24.44 ± 3.004	0.816		
SDF single selection^#^	22.07 ± 3.31	22.00 ± 2.95	0.959		

Comparative evaluation of relative sperm telomere length after each selection procedure

There were no statistically significant differences among the 23 infertile patients between the raw semen specimens, the processed samples, and the single selected spermatozoa in terms of relative STL (Figure 2B). The relative STL of both morphology categorizations was evaluated. The pairwise comparison of relative STL showed no remarkable differences in terms of relative STL after each selection procedure in the morphology ≤1% group (Table 4). Nonetheless, the pairwise comparison of STL showed that the mean RTL of the spermatozoa selected by the embryologist was statistically higher than the raw semen specimen (P=0.014) in the morphology 2% group. This increase in the RTL of the selected spermatozoa compared to raw semen should be attributed to both the selection procedure (processing sperm with DGC and single selection at ×400 magnification by the embryologist).

**Table 4:** Comparative evaluation of STL between and within two morphologies of severe teratozoospermia before and after semen preparation with DGC and single selection of spermatozoa cells. ^a^Statistical analysis was done based on the Mann-Whitney U Test. ^*^Pairwise comparison of STL between semen and single sperm selection was statistically significant within the morphology group of 2%. ^#^Single selection: Population of spermatozoa cells selected under ×400 magnification by an embryologist. STL: sperm telomere length, DGC: density gradient centrifugation, IQR: interquartile range.

Morphology					
STL/group	Group 1		Group 2		P-value
	Median	IQR (percentile 75-25)	Median	IQR (percentile 75-25)	
STL semen	0.59	1.57 – (-0.80)	0.36	1 – 0.15	0.926^a^
STL processed sample	0.29	1.26 – (-0.57)	1.22	1.58 – (-0.33)	0.369^a^
STL single selection^#^	0.30	0.95 – (-0.28)	1.74	2.35 – (0.46)	0.062^a^
P-value	0.807		0.000^*^		

Correlation between relative telomere length and sperm DNA fragmentation

The relationship between STL and DNA fragmentation was investigated using Pearson’s correlation. An aliquot of semen sample was used to evaluate DNA fragmentation and 5 ng DNA per µl was used in order to evaluate the correlation between STL and DNA fragmentation. There was no statistically significant association between STL and DNA fragmentation in the semen samples with severe teratozoospermia (r=0.01, P=0.964).

## Discussion

Sperm quality plays a crucial role in achieving favorable pregnancy outcomes during assisted reproductive technology (ART) treatments. Semen samples exhibiting severe teratozoospermia display high levels of DNA integrity defect [22,23]. Such patients may benefit from increased investment of time in sperm selection. The results of this study indicate that sperm selection at ×400 magnification can result in choosing spermatozoa with lower DFI from processed samples in severe teratozoospermia patients. The reduction rate of DNA fragmentation was estimated at 10%.

Although no improvement in relative STL was observed, the mean RTL of selected spermatozoa by embryologists was increased by 30% in the morphology 2% group. This result is in accordance with Lafuente’s findings [23]. Lafuente et al. (2018) used quantitative fluorescence in situ hybridization (q-FISH) with peptide nucleic acid (PNA) probes to evaluate the telomere length of spermatozoa before and after sperm selection by swim-up or DGC procedure [23].

Three previous studies have reported a positive impact of sperm selection on the telomere length of spermatozoa following DGC or swim-up techniques in semen samples [13,24-25]. Yang et al. (2015) and Zhao et al. (2016) conducted studies on patients with normozoospermia semen samples and evaluated STL with quantitative polymerase chain reaction (q-PCR) [13,24]. Also, Santiso et al. (2010) conducted a study on patients with heterogeneous semen sample criteria [25]. Infertile patients with normal and abnormal semen parameters were entered into Santiso’s study and their DFI was lower than in our study (based on DFI shown in Figure 1) [25]. Telomere length was measured by q-PCR in these three studies.

Lafuente et al. (2018), indicated that there was no improvement in telomere length after sperm selection by DGC and swim-up [23]. Also, the differences in their results were attributed to the different procedures used to assess STL [23]. Meanwhile, we used q-PCR to evaluate STL and our findings were similar to Lafuente’s result. These differing results may be attributed to the quality of semen samples used in the various studies. In cases of severe teratozoospermia compared to semen specimens, sperm selection via DGC does not appear to yield a population of processed spermatozoa with longer telomeres.

The coexistence of structural subtle defects of sperm heads with altered distribution of telomere sequences can be considered as the predictive factor for male factor infertility [6]. Also, there are markedly STL heterogeneity among individual spermatozoa within an ejaculate [11]. Therefore, motile sperm selection with proper morphology can enable embryologists to select spermatozoa with long telomere length.

Another finding of our study was that sperm selection from processed sperms led to spermatozoa with lower DNA fragmentation; however, TL was not higher in the selected spermatozoa compared to the processed samples in cases of severe teratozoospermia. In the subdivided groups, this result was observed in spermatozoa selected by the embryologist under conventional magnification in the ≤ 1% morphology group; however, compared to raw semen specimen, sperm selection under conventional magnification, combined with semen preparation by DGC, enhanced the embryologist’s ability to select spermatozoa with longer telomere length in the 2% morphology group. This significant evidence suggests that processed spermatozoa exhibit high homogeneity in terms of TL in severe teratozoospermia samples.

Therefore this result suggests sperm selection according to morphology at conventional magnification cannot be considered as a marker to predict STL in severe teratozoospermic samples.

While sperm selection by the embryologist at ×400 magnification can yield spermatozoa with lower DFI than processed sperms, this selection process should not be overly time-consuming. Sabour et al. (2022) found that prolonged exposure of human spermatozoa to PVP in patients with normal semen parameters has detrimental effects of PVP on sperm viability, morphology, and chromatin structure [26]. Jenkins et al. (2016) reported that poor semen samples characterized by teratozoospermia or AT are associated with specific epigenetic alterations that negatively impact sperm viability [27]. To prevent further deterioration in the biological parameters of severe teratozoospermia semen samples, efforts should be made to select the most suitable spermatozoa in a short time.

Limitations of the study

As a result of the low number of participants, we did not find a relationship between STL and DNA fragmentation. Therefore, there is a need to conduct research with a greater sample size to clear interpretation of STL and DFI.

## Conclusions

In conclusion, the results of our study using qPCR suggest no remarkable differences in telomere length of selected spermatozoa compared to non-selected spermatozoa of the processed population. It can indicate low heterogeneity of STL in individual spermatozoa of patients with severe teratozoospermia. Although selecting motile spermatozoa with appropriate morphology does not guarantee obtaining a population of spermatozoa with longer telomere in severe teratozoospermia samples with morphology index ≤1, these patients can benefit from sperm selection at ×400 magnification by decreasing sperm DNA fragmentation. Compared to raw semen, sperm selection at conventional magnification can help embryologists choose spermatozoa with higher DNA integrity in morphology 2% index. Additionally, no correlation was observed between sperm DNA fragmentation and STL in severe teratozoospermia samples.
